# Towards a Singing Voice Multi-Sensor Analysis Tool: System Design, and Assessment Based on Vocal Breathiness

**DOI:** 10.3390/s21238006

**Published:** 2021-11-30

**Authors:** Evangelos Angelakis, Natalia Kotsani, Anastasia Georgaki

**Affiliations:** Laboratory of Music Acoustics and Technology (LabMAT), Music Studies Department, National and Kapodistrian University of Athens, 15784 Athens, Greece; nkotsani@corelab.ntua.gr (N.K.); georgaki@music.uoa.gr (A.G.)

**Keywords:** biomedical signal acquisition, singing voice, data processing, breathiness, electroglottography, vocal mechanism, respiratory transducer, fundamental frequency estimation

## Abstract

Singing voice is a human quality that requires the precise coordination of numerous kinetic functions and results in a perceptually variable auditory outcome. The use of multi-sensor systems can facilitate the study of correlations between the vocal mechanism kinetic functions and the voice output. This is directly relevant to vocal education, rehabilitation, and prevention of vocal health issues in educators; professionals; and students of singing, music, and acting. In this work, we present the initial design of a modular multi-sensor system for singing voice analysis, and describe its first assessment experiment on the ‘vocal breathiness’ qualitative characteristic. A system case study with two professional singers was conducted, utilizing signals from four sensors. Participants sung a protocol of vocal trials in various degrees of intended vocal breathiness. Their (i) vocal output, (ii) phonatory function, and (iii) respiratory behavior-per-condition were recorded through a condenser microphone (CM), an Electroglottograph (EGG), and thoracic and abdominal respiratory effort transducers (RET), respectively. Participants’ individual respiratory management strategies were studied through qualitative analysis of RET data. Microphone audio samples breathiness degree was rated perceptually, and correlation analysis was performed between sample ratings and parameters extracted from CM and EGG data. Smoothed Cepstral Peak Prominence (CPPS) and vocal folds’ Open Quotient (OQ), as computed with the Howard method (HOQ), demonstrated the higher correlation coefficients, when analyzed individually. DECOM method-computed OQ (DOQ) was also examined. Interestingly, the correlation coefficient of pitch difference between estimates from CM and EGG signals appeared to be (based on the Pearson correlation coefficient) statistically insignificant (a result that warrants investigation in larger populations). The study of multi-variate models revealed even higher correlation coefficients. Models studied were the Acoustic Breathiness Index (ABI) and the proposed multiple regression model CDH (CPPS, DOQ, and HOQ), which was attempted in order to combine analysis results from microphone and EGG signals. The model combination of ABI and the proposed CDH appeared to yield the highest correlation with perceptual breathiness ratings. Study results suggest potential for the use of a completed system version in vocal pedagogy and research, as the case study indicated system practicality, a number of pertinent correlations, and introduced topics with further research possibilities.

## 1. Introduction

Singing is the result of certain neuromuscular functions pertaining to the vocal mechanism of the human body. Singing voice acoustic and perceptual properties have been studied since Ancient Greek times, mainly by Aristotle, Aristoxenus and Dionysius of Halicarnassus, and subsequently by Porphyrios, Nicomachus, and Cleonides [1]. This domain’s study has been continuing, and a plethora of modern-day research works have been actualized regarding its anatomical, physiological, and acoustical properties [2], however there is still much to be uncovered [3].

One of the features that seems to require further investigation is singing voice overall quality assessment, which entails the quantification of individual perceptual voice attributes that define it, as “no agreed-upon method currently exists for objective measurement of perceived voice quality” [4] (p. 457). As a result, perceptual evaluation is still an important factor in both voice disorder assessment and measurement instruments result comparison, despite the extensive research realized and validity concerns [5]. The quantification and understanding of such qualitative measures is also important to vocal education, as well as to early diagnosis of voice misuse and prevention of relevant vocal health issues in professional singing voice users (singers, actors, music teachers, etc.).

In order to achieve a higher level of understanding of the singing voice, and to facilitate a more precise vocal pedagogy and rehabilitation, it would be useful to research and further uncover the correlations between the vocal mechanism parts’ biomechanical functions and their resulting perceptual vocal properties.

### 1.1. Singing Voice Acoustic Analysis

A common acoustic model of the vocal mechanism involves the distinction of three parts: (a) the power source, or exciter (breathing system), (b) an oscillator (vocal folds), and (c) a resonator/filter (vocal tract) [6]. The resulting output is the auditorily perceived vocal timbre, which is defined by the contents of the vocal spectrum. This spectrum can be described, in the time domain, as the convolution of the glottal (vocal folds) pulse signal and the vocal tract filter impulse response [7,8]. Such an independent source-filter model of the vocal instrument (and the subsequent described convolution result) can be an “appropriate simplification” [7] (p. 2733) of a more complex interactions system.

The voice audio signal is easily acquired through a microphone, and its analysis can reveal quantifiable evidence on a number of the vocal perceptual characteristics. Fast Fourier Transform (FFT) as well as Logarithmic analysis methods on the spectrum of this microphone signal (Cepstral Peak Prominence (CPP), Smoothed Cepstral Peak Prominence (CPPS), Mel Frequency Cepstral Coefficient (MFCC), etc.), provide us with the potential to evaluate glottal events and vocal tract resonances separately [9] (for a brief introduction to CPP and CPPS cepstral measures please refer to Section 3.4).

### 1.2. Multi-Sensor Singing Voice Assessment

However, a more precise study of the voice could be achieved through combinatory analysis of the above vocal sound measures in conjunction with the respective vocal mechanism kinetic procedures. Such a venture requires the employment of more complex systems. In a previous positional/review journalpaper, analyzing the reported decline of operatic singing quality, as well as the challenges and recourse of vocal pedagogy [10], we have suggested the need for development of research and education tools, utilizing multiple sensors to transduce data regarding primary kinematic and acoustic functions of the vocal mechanism. We also proposed that a series of experiments be actualized, aiming towards the clarification of biomechanical-acoustical correlations.

The aim of the present work Authors: Changed for text clarity is multifold: (a) the initial configuration of a modular, multi-sensor singing voice analysis system; (b) the application of that system’s pilot study to research aspects of the breathiness characteristic in singing; and (c) the utilization of the aforementioned research’s data for the evaluation of vocal breathiness possible effects on the accuracy of an under-development vocal tuner software.

The sensors incorporated to the above system were an Electroglottograph (EGG), two Respiratory Effort Transducers (RET), and a Condenser Microphone (CM), while its pilot study investigated possible system issues regarding: system and sensor usability, singer–user convenience/task-hinderance level, compatibility, connectivity, signal acquisition, data recording synchronization, and signal noise levels. The vocal breathiness characteristic in singing was examined through a multi-faceted scope. Evaluation measures used were Acoustic Breathiness Index (ABI), CPPS, vocal folds Open Quotient (OQ) —computed both with the Howard (HOQ) and the DECOM methods (DOQ)—, singing voice Fundamental Frequency ($f_{o}$), phonation duration, "respiratory management strategies" (i.e., participants’ individual respiratory management strategies in singing, through —either conscious or unconscious— muscular control of the breathing system), and breathiness level perceptual evaluation. A new multivariate index, CDH (CPPS, DOQ, HOQ), was also proposed.

As mentioned above, experimental data extracted during the above pilot study were also used to evaluate the efficiency of a microphone as sensor for pitch tracking purposes in the case of users with varying degrees of vocal breathiness. This was realized by assessing the deviation of Praat’s [11] pitch-tracking algorithm results between (1) CM signal and (2) the respective EGG signal, in ‘distinct levels of intentionally breathy singing voice’. This algorithm has been used in the $f_{habitual}$ frequency tool [12] and is intended for use in Vocal Tuner Tool, both being parts of the ‘Assistance for students in Singing and Music Aesthetics’ (ASMA) project, involving the authors of the present work.This new tool, estimating voice fundamental frequency in combination with various spectral features of the voice, is designed for use from school children and their teachers, without requiring the added presence of medical or vocal experts (perceptually detecting possible vocal breathiness prevalence of users). Results from a performance study on seven established pitch detection algorithms [13] have shown indications of a “pitch error” increase in pathological voices. Breathiness has been reported to have a 24.2% percent prevalence in a study of 71 children 3–9 years old [14], and 37.71% prevalence in a study with 70 children aged 6–10 [15]. It was therefore considered important to test the algorithm in breathy singing, and to compare its deviation between application on CM and EGG signals. It was hypothesized that an EGG signal could be able to provide more accurate fundamental frequency estimation data in cases of breathiness prevalence, as (a) breathy voice air turbulence noise is not a part of the EGG signal, (b) vocal tract ‘filtering’ on voice signal is not actively affecting the glottal pulse frequency, and (c) EGG signal is impervious to ambient noise, which could mask the acoustic voice signal during field experimentation with breathy voices (where pitch strength is already low [16]).

### 1.3. Related Work

Multi-sensor singing voice analysis is a field that is currently expanding. Examples of software for combined EGG and microphone signal analysis are ‘PhaseComp’ [17] and ‘VoceVista Video Pro’ [18], which have been developed by EGG device manufacturing companies. Additionally, the open-source voice analysis software Praat [11] has incorporated scripts for working with an EGG signal, while the twocompanion software ‘VoiceSauce’ [19] and ‘EGGWorks’ [20] can be fused to export combined analysis results for audio and EGG signals. Research on the singing voice realized using multiple sensor input is not very common. Watson and Hixon [21] studied opera singers respiratory kinematics using microphones, magnetometers, and video recording, while Salomoni et al. [22] used a microphone, respiratory inductance plythesmography bands, and a pneumotachograph. A 2018 study [23] compared measurements between an electret condenser microphone, a sound level meter, and a piezoelectric contact microphone, assessing (among others) pitch inaccuracies and cepstral measures of singing voice quality estimation in 14 professional singers. Furthermore, a section of the international UNESCO ‘iTreasures’ project for the preservation of ‘Intangible Cultural Heritage’ [24] resulted in the creation of a platform for the capturing and analysis of rare singing techniques (i.e., Corsican Paghjella, human beat box, and Byzantine chanting). This platform employed data capturing from a microphone, an EGG, an imaging camera, a breathing belt, an ultrasound module, and piezoelectric accelerometers [25]. Another study on singers employing both invasive and non-invasive sensors regarded the definition of “supported singing voice” through the comparison of the singers’ self-perception of supported and un-supported singing with quantitative measures [26]. Additional sensors that can be (or have already been) used for singing voice biofeedback include spirometers, skeleton tracking cameras, videokymograph, and functional magnetic resonance imaging devices [10].

Although the studies listed here performed a multi-sensor analysis, a significant number of researchers have studied the singing voice with the use of solely accelerometers, which can be employed for the examination of various voice-related measures, such as fundamental frequency, open quotient, glottal airflow [27], and subglottal pressure [28], that are traditionally captured with a microphone, an EGG, or airflow measurement devices. Accelerometers have also been used in ambulatory voice monitors for analysis of singers’ vocal qualities, vocal habits, phonotrauma risk assessment [29,30], and detection and classification of singing [31].

A recent study [32] on 20 professional singers used a videonasoendoscopic camera system, a laryngostroboscopic system, an EGG, and a microphone to “assess and quantify singers’ strategies for adding air to phonation to sound ‘breathy’ in a healthy manner” [32] (p. 1). Finally, the study utilizing the set of sensors closest to the one described in the current work was published by Ternström et al. [33], and reported findings from eight trained female singer regarding the effects of relative lung volume on the EGG waveform.

Vocal ‘breathiness’ is a qualitative vocal characteristic that has been studied quite extensively during the last decades [32,34,35]. It was chosen as an assessment case study due to (a) its association with all three parts of the vocal mechanism, (b) its connection to overall voice quality [36], and (c) its prevalence in children with dysphonia, a factor which the authors wish to control for in ASMA project. The Consensus Auditory Perceptual Evaluation of Voice (CAPE-V) standardized clinical protocol definition of the breathiness vocal attribute is “audible air escape in the voice” [37] (p. 127), while the ABI introductory study [35] specifies the characterization of breathy voice by a “turbulent noise during phonation with excessively high frequency resulting from air leakage during glottal closure” (p. 511.e11). This attribute is of interest to vocal pedagogy [32] and linguistics [38], as it is a quality-defining perceptual characteristic [34], but also important to phoniatrics, as it has also been connected to pathological conditions, such as vocal nodules, vocal fold bowing, paralysis or paresis of the recurrent laryngeal nerve, and acute laryngitis [35]. Breathiness has long been reported as “quantitatively related” to insufficient vocal fold closure [39] (p. 5), resulting in continuous air leakage through the glottis (i.e., the opening/space between the two vocal folds). Abduction (opening) and adduction (closing) of the vocal folds is a determining factor for phonation and text articulation [6]) during phonation. This makes breathiness a particularly important characteristic, as insufficient vocal fold closure may imply a medical condition [40] but could also result in one [41]. Despite the apparent multidisciplinary interest in the ’breathy’ voice quality, there are still publications reporting a lack of adequate data correlating the ’breathy voice’ perceptual, qualitative feature, to the pertinent biomechanical functions and the resulting acoustical, quantitative measures [32].

A ‘breathy’ voice may be tolerable, or even desired as a timbral option in some music genres, such as Jazz, or some 20th–21st century music compositions using ‘Extended Vocal Techniques’ (a term that is used to include phonation modes or experimental techniques that deviate from the norm for Western classical singing, such as multiphonics, overtone singing, ingressive singing, growling, etc.) [42] (pp. 21–22). This intentional use of the breathiness characteristic is sometimes referred to as “air added to the voice”, to distinguish it from involuntary breathy voice production [32] (p. 1). This study makes use of the term breathy as the perceptual characteristic, without differentiating between these conditions. Unintentional vocal breathiness is usually considered as a negative characteristic in many music styles, such as operatic singing [43]. This is due to breathiness being connected to relatively low vocal intensity (opera singers have to be clearly audible in large auditoriums over the sound of a symphonic orchestra without amplification) and low “pitch strength” [16], the latter implying the presence of a wide-band noise [44] from the leaking air. Prevalence of this noise indicates the breathiness perceptual degree and has been studied using various acoustic measures [35].

The following is a brief overview of the related work pertaining to vocal breathiness analysis. Pongweni corrected typo in name [45] studied the effect of breathy voice on Shona vowels using the amplitude, pitch and formants. Fritzell et al. [39] concluded that a quantitative relationship exists between breathy voice quality and glottal insufficiency in pathological voices according to perceptual judgments. Scherer and Titze [34] extracted the abduction quotient ($Q_{a}$) from electroglottographic recordings in speech and singing tasks in different voice qualities (breathy, normal, and pressed or constricted), concluding that the abduction quotient decreases from breathy to pressed voice and suggesting that it corresponds to the effective glottal width. Hartl et al. [46] showed that unilateral vocal fold paralysis (UVFP) related breathiness was best correlated with airflow measurements and that 10 of the 14 objective acoustic and aerodynamic parameters successfully distinguished patients with UVFP from the control group. Shrivastav and Sapienza [47] compared several measures obtained from the output of a previous auditory model to the perceptual ratings of breathiness (for the Kay Elemetrics Disordered Voice database) using multidimensional scaling techniques, concluding that the perceptual ratings of breathiness were best predicted by the partial loudness of the periodic signal and stating the importance of an auditory model as a signal processing front-end in obtaining objective measures of voice that correspond closely with listeners’ perception of breathiness. Teixeira et al. [48] presented a non-invasive procedure for automatic diagnosis of pathologies of the larynx using jitter, shimmer (correlating it with breathiness), and HNR parameters, and proposed threshold values for pathological voices. Fraile and Godino-Llorente [49] highlighted the reliability of CPP in dysphonia detection and its extended use to the evaluation of overall voice quality, commenting, however, that this particular measure is not sufficient for predicting voice quality aspects related to breathiness.

Borsky and Guðnason [50] classified five different voice types (modal, breathy, rough, soft, and pressed) with Gaussian Mixture Model (GMM) from speech samples using an electroglottograph and extracting the MFCCs. Barsties v. Latoszek et al. [35] proposed a nine-variable acoustic model for the multiparametric measurement of breathiness, the Acoustic Breathiness Index (ABI), in dysphonic voices using stepwise multiple regression analysis. Kadiri and Yegnanarayana [51] classified breathy, modal, and tense voice using support vector machines (SVMs) and the zero-time windowing method, optimizing the detection of phonation type when combining various acoustic and glottal features. Aaen et al. [32] assessed air added to the voice (i.e., a singer’s strategy of adding air to create a vocal breathiness effect in a healthy voice) by extracting various features from laryngostroboscopic imaging and electroglottograph data, and observed a glottal gap along the edge of the length of the vocal folds, as well as significant differences in various acoustic parameters. Tylečková and Skarnitzl [52] proposed quantitative ranges of voice quality parameters on non-pathological voices based on speech reading tasks in Czechs, concluding that the estimated CPP values describe modal (e.g., non-breathy) phonation. Barsties v. Latoszek [40] quantifyied evidence for the diagnostic accuracy of ABI, in terms of sensitivity and specificity, from 34 research works, confirming the ABI’s robustness and validity. Murton et al. [53] used CPP to evaluate clinical voice concluding in CPP cutoff thresholds indicating the presence of voice disorders.

A recent publication indicated that breathiness studies have been (a) mostly concerned with speech, (b) included singers as participants in a percentage seemingly lower than 2% (35 out of 1923 in the 29 reviewed studies), and (c) rarely involving female and male singers concurrently [32].

The system-in-development described and tested in the present publication will be, when completed and to the best of our knowledge, one of the few non-invasive tool attempts for the voice to combine acoustic, glottal, and respiratory signal acquisition from dedicated sensors. The assessment experiment and analysis included measures that are rarely studied against each other. A strong correlation has been hypothesized between perceptual breathiness and CPPS, as well as between breathiness and OQ. The Acoustic Breathiness Index was computed and a new multivariate regression model (CDH) was introduced, to associate data results from CM and EGG signals for breathiness prediction, while the possibility of combinatory analysis, using ABI and CDH, is also explored.

Reports of transglottal airflow increase up to ~60% in breathy vowel phonation [54] also lead to the assumption of a decrease in maximum phonation duration with breathiness perceptual degree.

Furthermore, following the indications for pitch detection algorithm accuracy comparison between healthy and pathological voices [13], a secondary hypothesis was made. This predicted a non-significant deviation when comparing results from the Praat pitch detection algorithm between application to EGG and microphone signals of non-breathy singing, but a significant deviation in respective results for breathy singing.

Correlation analysis appeared to confirm the aforementioned hypotheses, disproving only the hypothesized accuracy decrease in microphone signal fundamental frequency estimation in breathy singing, when compared to an EGG signal.

The innovative aspects of this work consisted mainly of (i) the design and testing of a developing multi-sensor architecture to capture various parameters of the singing voice; (ii) the comparative study of a specific pitch detection algorithm effectiveness in CM and EGG signals in increasing singing voice breathiness conditions; and (iii) the introduction of CDH, which is a new multi-variate regression model for the prediction of breathiness in the singing voice, combining data from both microphone and EGG signals, which revealed significant results for our dataset.

## 2. Materials and Methods

The system setup described here is being designed as a portable, non-invasive, modular monitoring system for signal acquisition, synchronization, and offline processing of multiple sensors, transferring data from all three main parts of the vocal mechanism (breathing system, phonatory system, and vocal tract). A distinct type of sensor was used for each of the above mechanism parts. The exception to this was the monitoring of the vocal tract with the use of a microphone, which can record the final vocal output, resulting from vocal tract filtering on the vocal folds pulse signal. However, as mentioned above, microphone signal data analysis can reveal useful information distinctive of the vocal tract modifications and filtering. All sensors used in this initial system version could sent data for recording and processing independently, through their respective signal acquisition units, without direct sensor inter-communication.

**Figure 1:**
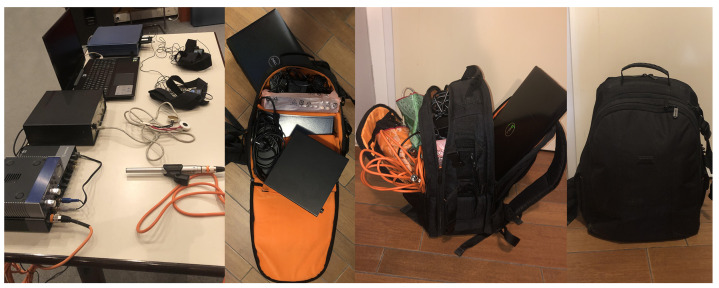
The entire experiment setup equipment, both ready-for-use (**left**) and fitting a backpack (**center** and **right**).

As portability was a primary prerequisite for the equipment choice, the entire experiment equipment (excluding the microphone stand) was fitted and held in a large photography backpack, as shown in Figure 1. This will allow for future field experiment measurements to be conducted.

### 2.1. Breathing System Sensors

The breathing system was monitored by two respiratory effort transducers (Biopac SS5LB) connected to a Biopac MP35 Signal Acquisition Unit (Biopac Systems, Goleta, CA, USA). Each of the transducers was connected to a soft, adjustable nylon strap and measured the circumference expansion and contraction of a certain torso region. They were selected for the experiment as they claim to “measure extremely slow respiration patterns with no loss in signal amplitude while maintaining excellent linearity and minimal hysteresis” [55]. Additionally, SS5LB belts are lightweight, extremely unobtrusive for the singers, and sterilizable. One strap was placed around the thoracic region (RETt), about 5 cm underneath the armpits (as suggested by the manufacturer), and a second was placed around the abdominal region (RETa), right under the navel. Following the SS5BL Instruction Manual, the straps were adjusted to have a slight tension at maximum expiration state for each participant. RET placement also followed the respective model used by Watson and Hixon [21].

### 2.2. Phonation

**Figure 2:**
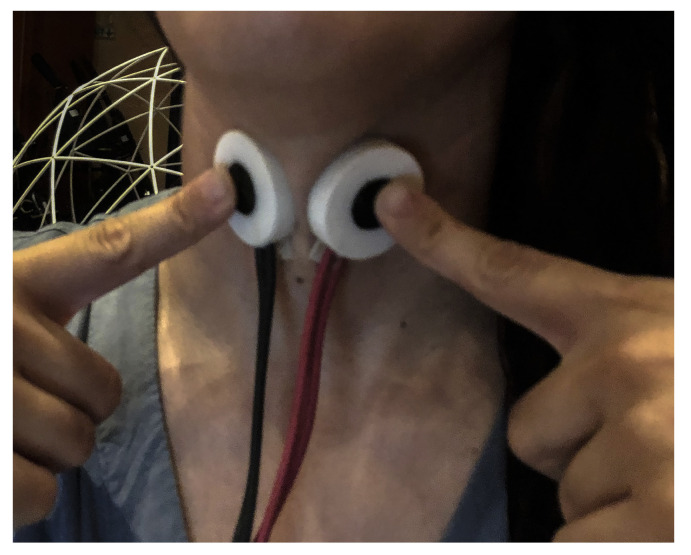
Participant displaying EGG electrode placement and alignment.

The vocal folds’ behavior was monitored with the use of an Electroglottograph (EGG). The EGG is a non-invasive sensor that has been used extensively during the last six decades for studying various aspects of laryngeal voice production, and its application has resulted in a number of contributions to the science of the singing voice [56]. It employs the use of two electrodes, which are placed bilaterally on the larynx (usually with the help of an external soft strap). The EGG provides output regarding the relative contact area of the vocal folds [57] in real-time, by measuring electric conductivity of a small current, with a frequency commonly ranging from 300 kHz to several megahertz [58]. For the present experiment, the EG2-PC electroglottograph by Glottal Enterprises was used. This model additionally employs a ‘Laryngeal tracking indicator’, which can be utilized to ensure correct electrode placement and minimize vertical larynx movements’ effect on performance [57]. The unit is powered with the help of two internal rechargeable batteries and remains unplugged during experimentation to allow for maximum line noise reduction and transportability. To achieve optimal signal acquisition and minimize noise, the EGG was used, making sure to maintain a small interelectrode angle and distance (as shown in Figure 2), following the guidelines by I. Titze [59] and the instructions for the EGG model used in our experiment by A. Michaud, including methods by N. Henrich and B. Gautheron [60]. The latter instructions were also followed for experimental conditions, device settings (with the exception of setting the EGG output level to ‘High’), device use, and electrode alignment, whereas electrodes were held in place only by hand to allow for maximum vocal freedom.

### 2.3. Vocal Output

The Behringer ECM8000 measurement microphone was use to capture the vocal output. It is an omnidirectional microphone with flat frequency response at a 15 Hz to 20 kHz range and requires phantom power (+15 to +48 V). The microphone was placed at a 40 cm distance from the singer’s mouth and its signal was passed through a PreSonus TubePre professional microphone tube preamplifier, using XLR cables.

### 2.4. Connections

Both the EGG signal and the pre-amplified microphone signal were input to two distinct channels of a Steinberg UR44 external sound card. The sound card was connected to a dedicated Dell G5 15 Laptop via a USB Type-C cable.

### 2.5. Software and Sensor Signal Recording

**Figure 3:**
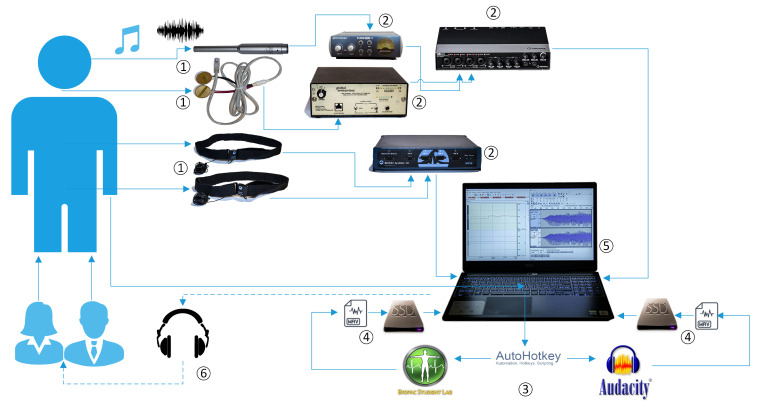
System Architecture: ➀ Sensors. ➁ Signal Acquisition and Amplification Units. ➂ Data Recording Synchronization. ➃ File Export and Storage. ➄ Data Analysis. ➅ Post hoc Perceptual Evaluation.

The (a) microphone audio and (b) EGG input signals were recorded at 48 kHz/24 bit PCM (Pulse-Code Modulation audio format), following the suggestion of a previous study [32] for a minimum of 16 kHz recordings, and using the Audacity Open Source multi-track audio editor and recorder for Windows (Version 3.03). The resulting audio was exported in files with .wav extension. The two Biopac SS5LB sensors signal, passed through a Biopac MP35 Acquisition Unit, which output the acquired signals to the same laptop via USB connection, as depicted in Figure 3. This was then recorded with the use of the Biopac Student Lab (BSL) 4.1 Pro Software, using the software default sensor settings (not the SS5BL settings). Waveforms from SS5LB sensors signal were also exported in .wav files to allow for maximum compatibility in analysis with the microphone and EGG signal files. In order to ensure the optimum synchronization of the four sensor signals, being recorded by two distinct software programs, a script was written in the open-source scripting language AutoHotkey (version 1.1.33.09). This enabled recording to commence in both programs simultaneously (using a single keyboard key), and to thus result in exported files with common time-stamps that could be synchronized more accurately for the purpose of data analysis.

### 2.6. Ambient and Hardware Noise

All experiment measurements took place in a soundproof recording room at the recording studio of the Laboratory of Music Acoustics and Technology (LabMAT) of the Music Studies Department of the University of Athens. In order to obviate a substantial portion of possible interference and noise during measurement recordings, a laptop computer was setup to record using internal battery power and optimized for minimum-to-zero fan noise. Furthermore, the external sound card was powered from the laptop using a USB-C connection, while the EGG processor was powered by two internal batteries. The EGG manufacturer claims that their “electroglottographs produce very low-noise EGG waveforms” (https://www.glottal.com/Electroglottographs.html (accessed on 27 November 2021)). All three devices were disconnected from external power sources and current transformers.

**Figure 4:**
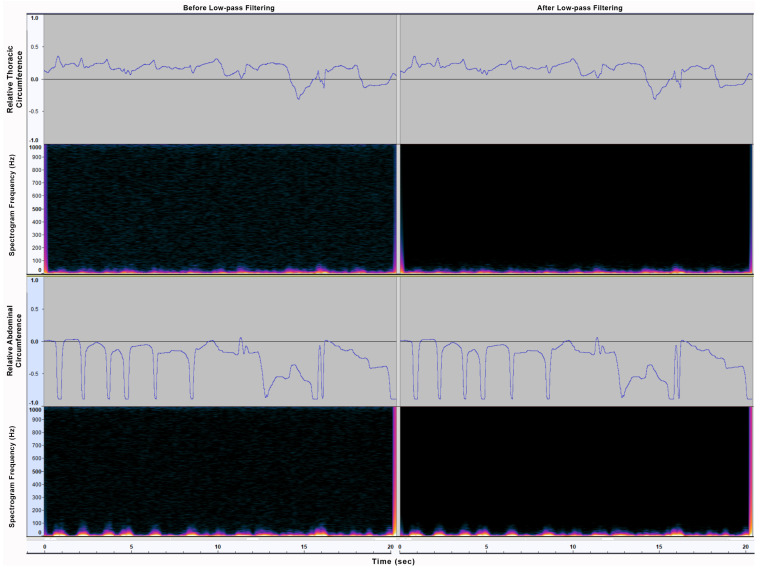
Waveforms and spectrograms of the two (**top** and **bottom**) RET sensors’ signal low-pass filter application for noise removal, in a sample of intentionally excessive muscle movement (**Left**: before filtering; **Right**: after filtering).

The Signal-to-Noise Ratio (SNR) was 45 dB for the recorded CM signals and 30 dB for the EGG signals. This should allow the extraction of valid EGG results (SNR ≥ 30 dB) and CM results with a theoretical measurement accuracy of 99% (SNR ≥ 42 dB), according to reference studies [61,62]. For the purposes of this particular study, the RET signal was analyzed qualitatively through signal waveform, where no noise seemed to visually interfere. Due to the nature of respiratory muscles movement and relatively very low speeds, RET signal frequencies of interest reside in the range of under 10 Hz (normal respiratory cycles have a frequency of approximately 0.2–0.3 Hz, while frequencies higher than 1 Hz “are commonly registered during high-intensity exercise” [63]). For system test purposes, a software low-pass filter [64] was applied in Audacity, using a relatively high threshold of 40 Hz, in order to overcompensate for any excessively fast muscle movement, but cutting off possible interference of local electrical current (50 Hz). This filtering resulted in elimination of any noise from mechanical or electrical sources upwards of 40 Hz, as shown in Figure 4.

### 2.7. Experiment Participants

Professional operatic singers have been used as participants in research studies pertinent to the singing voice, as their extremely demanding profession [10] requires extensive training and practice. The genre generally known as “operatic singing” or “Western classical singing” is furthermore one of the most demanding types of vocal music. It encompasses a huge variety of sub-genres of many Western vocal traditions, ranging from the 17th century to today. It requires singers to employ a high skill level pitch precision, vocal range, volume/audibility, lyrics discernibility, voice health and stamina, vocal agility/flexibility, musicality, stylistic proficiency, acting capability, etc. It is a trait often compared to elite athleticism. This has been reported to promote a higher degree of kinesthetic control [65] and enable the voluntary isolation or prevalence of distinct feedback modalities (auditory, kinesthetic/somatosensory) [65,66]. Two participants took part in this study (1 female, 1 male—authors NK and EA, respectively). Both were professional singers with classical training and professional experience (21 and 28 years, respectively) both as singers and teachers of singing. Participants stated that they perceived their vocal instrument to be in good health and that, in the past, they have both had stroboscopic medical evaluations that had revealed no chronic vocal health issues. The data are available in a publicly accessible repository (https://github.com/nataliakotsani/Singing-Voice-Multi-Sensor-Analysis-Tool/tree/main/DATASET (accessed on 28 November 2021)).

### 2.8. Experimental Protocol

The participants used the recording room one at a time. Participants were allowed to commence at their own convenience and follow the protocol at their preferred pace, pausing between trials, as they needed. The protocol of trials was discussed and was available for them in a printed page. The protocol consisted of vocal trials on distinct voluntary controlled degrees of vocal breathiness in singing. Three breathiness cases were studied: (1) Non-Breathy singing Voice (NBSV), (2) Breathy singing Voice (BSV), and (3) ‘Gradual’ Breathiness singing Voice (GBSV), where the breathiness characteristic had to slowly and gradually be changed from non-breathy to breathy, quantifying data for the varying degrees of this perceptual voice feature. Each of the above cases were recorded on three degrees of vocal intensity (medium, high, low—m, h, l, respectively), resulting in a total of nine experimental conditions. For each of these nine conditions, the participants were asked to sing the following six trials, sustaining their sound for as long as it was comfortable for them, aiming towards their maximum duration at optimum vocal efficiency. Trials were (a) single sustained note on a comfortable tone, (b) single sustained note on a high tone, (c) single sustained note on a low tone, (d) ascending vocal glissando (2-octaves minimum), (e) descending vocal glissando (2-octaves minimum), and (f) ascending and descending 2-octaves arpeggio. This resulted in a total amount of 54 distinct trials for each participant. Each participant’s complete trial run was recorded as one incessant file, including failed and repeated trials. Participants were instructed to cough lightly employing a simultaneous engagement of the abdominal region muscles before commencing the trial run, as well as right after concluding the last trial. This served the purpose of post hoc data synchronization via actively generated common events, which is reported in the literature as a common approach [67]. Participants stated orally, for the recording, the exact condition for each trial before commencing it.

## 3. Analysis

The section that follows describes in detail the way in which the experiment data was handled, rated, classified, and subsequently analyzed (a) quantitatively, (b) qualitatively, and (c) statistically.

### 3.1. Data Handling and Analysis Parameters

Total experiment time for participants 1 and 2 (P1 and P2) was 23 min 14 s and 16 min 42 s, respectively. Experiment data were exported into 48 kHz/24 bit .wav files and processed in Audacity. Audacity inherently performed lossless files conversion from 24-bit integer to 32-bit floating-point. Initial file processing involved separation of 32-bit stereo audio files into two separate 16-bit monophonic files (one for each sensor) and the subtraction of aborted trials, silent intervals, and oral trial descriptions, resulting in concatenated .wav audio files containing solely the experiment trials sound. Processed files had respective duration of 7 min 31 s (P1) and 7 min 44 s (P2) and were subsequently segmented each into multiple 2 s consecutive numbered .wav sample files, preserving the initial 16 bit-rate. Sample files were numbered from 000 to 224 for P1 and from 225 to 455 for P2. Microphone and EGG sound channels were processed simultaneously, creating an equal number of audio files for the EGG signal.

For the analysis, the dataset samples were considered as independent data points (as in corresponding studies of the literature [68]), instead of the subjects. The descriptive outcomes and the correlation factors, between the selected parameters and the perceptual breathiness ratings, were extracted using specific statistical libraries in python programming language, and the implementation is available in a public repository (https://github.com/nataliakotsani/Singing-Voice-Multi-Sensor-Analysis-Tool, (accessed on 28 November 2021); https://doi.org/10.5281/zenodo.5732933 (accessed on 28 November 2021)) [69].

The selected features for the analysis were the Pitch Difference between estimations from the CM and the EGG signals, in both Hz (PD) and Mel Scale (MPD), the Smoothed Cepstral Peak Prominence (CPPS), the Open Quotient extracted with both the derivative of the EGG signal-DECOM-(DOQ) [70] and Howard (HOQ) [71] methods, as well as the Acoustic Breathiness Index (ABI) [35].

The correlations between the perceptual breathiness ratings and the above parameters were analyzed. Of these acoustic and glottal parameters, the ones that displayed higher correlations to breathiness ratings were CPPS, DOQ and HOQ. These were selected in an attempt to model the relationship between them and perceptual breathiness. Using multiple linear regression, a new index (CDH) for the prediction of the breathiness rating was computed using the microphone and EGG signal results, through exclusively the CPPS, DOQ, and HOQ parameters. In addition, the CDH index was experimentally combined with the ABI index, a combination that yielded the highest correlation coefficients for our dataset.

### 3.2. Perceptual Evaluation

The above 456 two-second microphone audio sample files were evaluated perceptually, using the same two experiment participants as judges, utilizing their expertise and experience as singing teachers [72]. The evaluation was performed using a 9 point discrete rating scale from ‘0’ (absolutely no perceivable breathiness in the voice) to ‘4’ (excessively breathy vocal sound with a barely discernible pitch), including half point values (0.0, 0.5, 1.0, ... , 3.5, 4.0). Both judges reported to have no permanent or impermanent hearing or vocal impediment. They evaluated all 456 samples (from P1 and P2), but each in a distinct randomized order, using separate online forms, to avoid influence for consecutive samples and priming effects, as well as to control for participant fatigue effect distribution.

Each participant evaluated the samples in four approximately 1-h sessions, with interposed intervals to further control for fatigue, using the same model of closed type headphones, and each at their own, personal environment.

The double attribute of the participants/raters was decided in order to ensure ability of the raters to perceptually discern between variable conditions of vocal breathiness. A previous intra-rater reliability and validity study on non-expert dysphonic patients [73] reported findings that self-rating of perceptual voice qualitative features (including breathiness) appeared to be consistent and partially valid. Partial validity in that study [73] was hypothesized to be attributed mainly to self-raters’ lack of expertise, poor auditory discrimination and poor auditory memory skills prevalence in vocal patients, as well as psychogenic factors. These influences were considered to be non-existent in current research with healthy, expert participants.

In the work presented here, self-rating was predicted not to be significantly affected by biases as (a) audio samples were merely 2 s long, leading towards judgment for the specified vocal property, rather than overall singing quality; (b) during the recording experiment, participants were asked to sing trials utilizing various degrees of breathy voice (thus minimizing the response bias for a ‘good’ rating from themselves as judges); (c) samples were presented for judgment in separate computer-generated randomized orders, to disassociate them of any sense or knowledge regarding the intended target breathiness degree for each sample; (d) trial audio segmentation in 2-s samples, the large total of samples number (456), and a 15-day interval between measurements and perceptual judgment, was employed to help eliminate any associative memory between experiment trial expectancy and ratings; and (e) the judges had knowledge that participants’ initial breathiness target degree was irrelevant to the study (as breathiness is a perceptual characteristic), that correlations between intended voice breathiness degree and perceptual rating was not being examined, and that statistical analysis would be based solely on the auditory rating and not initial target intention.

Evaluations comparison between judges for the total 456 samples, in the 9 point scale, revealed absolute judgment agreement in 63.8% of the samples (*n* = 291), 1 point judgment deviation in 22.8% (*n* = 104), 2 to 3 point deviation in 11.9%, 4 to 5 point deviation in 1.5% (*n* = 7), and 0% for 6 to 8 points deviation.

**Figure 5:**
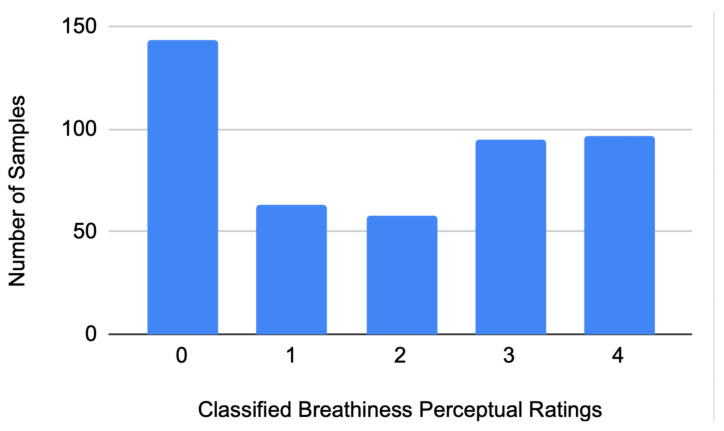
The numbers of samples per class.

For the descriptive statistics outcomes, the above samples were classified according to their average breathiness perceptual ratings. Calculating the average from the two judges for each sample resulted in scores with a total of 17 values and a 0.25 step (0.00, 0.25, 0.50, 0.75, ..., 3.75, 4.00). For comparability with previous studies that used a 5-point evaluation [32,47], samples were then classified using a taxonomy of the above 17 values into five classes (0, 1, 2, 3, 4) of three to four values per class, minding the first and last classes (0 and 4, respectively) to include exactly three values each. The latter was opted in order to achieve a more uniform distribution of the sample population (see Figure 5), as the experimental breathiness target conditions were (a) NBSV (which should yield samples belonging mainly to class 0), (b) BSV (which should yield samples belonging mainly to class 4), and (c) GBSV (which should yield samples belonging to all classes, including 0 and 4). Consequently, the grouping used for the five respective classes was <0.75, ≤1.5, ≤2.25, ≤3.25, ≤4.

### 3.3. Pitch Detection in Microphone and EGG Signals

The aim of this experiment included the assessment of the Praat pitch detection algorithm, used also in the ASMA project for the $f_{habitual}$ frequency tool [12]. In previous works regarding the comparison between the microphone and the EGG signals, Vieira et al. [74] compared the acoustic and EGG signal jitter for dysphonic speakers, and Jang et al. [13] studied the comparative results of seven pitch detection algorithms for verification of adequacy in pathological voices.

For the comparison of the pitch detection algorithm between microphone and EGG signals we used our dataset and the python’s parselmouth library [75] which provides an interface to the internal Praat [76] code, directly accessing Praat’s C/C++ code. The Praat’s pitch detection algorithm was used in the microphone and EGG signals, comparing the differences of the results as the breathiness evaluation values increased. A correlation analysis was conducted between the breathiness perceptual ratings and (i) the difference of the pitch detection algorithm results when applied over the CM and the EGG signals, and (ii) the absolute values of that same difference.

### 3.4. CPPS of the CM Signal

For the extraction of the CPP, a Fourier transform is applied to the logarithm power spectrum of a recorded sound wave. The relative amplitude of the cepstral peak in relation to the expected amplitude of the cepstral peak is then estimated, using linear regression. Intuitively, it has been mentioned that the CPP represents the degree of periodicity in the voice signal and higher CPP values emerging from well-defined harmonic structure [77], but, despite its extended use for overall voice quality evaluation, a definite explanation of “what CPP actually measures” [49] is still lacking.

Formally, given a signal s(t), its real cepstrum, or power cepstrum, is equal to the Fourier transform of the logarithm of its power spectrum, according to the first definition of cepstrum [49]

(1) $$C_{r}\left(q\right)={F\{\log|S\left(f\right)|}^{2}\}$$

where $S^{2}\left(f\right)$ is the power spectrum of the signal

(2) $$S^{2}\left(f\right)=F\left\{E\left\{s\left(t\right)\times s\,\left(t-\tau \right)\right\}\right\}$$

A variant of CPP, called smoothed CPP (CPPS), was selected for acoustic analysis in the present work. This variant utilizes smoothing operations added both in temporal and cepstral domains [38], and has been reported to provide higher correlation with breathiness [49]. More specifically, the modification of the CPP algorithm consisting of an additional processing step, smoothing the individual cepstra (before extracting the cepstral peak and calculating the peak prominence), in two steps, as, first the cepstra are averaged across time, and second, the running average of cepstral magnitude is calculated across quefrency.

The CPPS was extracted through Praat software (version 6.1.41) [76], with the same parametrisation as Maryn’s and Corthals’ Acoustic Voice Quality Index (AVQI) Praat script (v.02.03) [78,79], i.e., for the Power Cepstrogram, pitch floor: 60 Hz, time Step: 0.002 s, maximum frequency: 5000 Hz and pre-emphasis from 50 Hz, and the CPPS where extracted without subtracting trend before smoothing, with time averaging window: 0.01 s, quefrency averaging window: 0.001, peak search pitch range: 60–330 Hz, tolerance: 0.05, parabolic interpolation, trend line quefrency range 0–0.001 s, straight trend type and robust fit method.

### 3.5. OQ of the EGG Signals

The contact or closed quotient (CQ) is used to compare the duration of the contact phase to the period of the vibratory cycle. It is complementary to the open quotient (OQ), since CQ and OQ ratios together constitute the total glottal cycle, and therefore CQ + OQ is always equal to 1. DECOM is an algorithm to calculate the OQ based only on the first derivative of the EGG signal (DEGG). This algorithm was proposed by Henrich et al. [70] and extracts the OQ using a correlation-based method to estimate the distance between two peaks of the dEGG signal corresponding to two respective consecutive vocal folds closing instants, as well as the distance between two peaks corresponding to respective consecutive instants of vocal folds opening and closing. Howard [71] proposed a different method in which the contacting event is defined by the peak in the DEGG signal during the closing phase, while the closed phase is defined by linear quantization between samples either side of a 3:7 threshold.

For the OQ computation, a Praat script extracting OQ with both DECOM and Howard methods was used [80] and the average value for each sample with both methods was extracted.

### 3.6. ABI of the Microphone Signals

Barsties v. Latoszek’s et al. multivariate acoustic model for the evaluation of breathiness [35] is more suited to dysphonic voices, as it requires both concatenated voice samples of continuous speech and a sustained vowel. In the present work, focusing mainly in evaluating intentionally breathy singing phonation, an attempt to attain deliberate continuous speech samples in various pitch regions and intensities, fully compliant with their respective GBSV samples, would probably produce disputable results. For this reason, the same sustained vowel file was given as an input for both the continuous speech and the sustained vowel sample, accepting the possibility of model inaccuracies, and therefore interpreting results with care.

For rating a pathological voice’s breathiness Barsties v. Latoszek [35] proposed a combination of nine acoustic variables using stepwise multiple regression analysis: ABI = (5.0447730915 − [0.172 × CPPs] − [0.193 × Jit] − [1.283 × GNEmax − 4500 Hz] − [0.396 × Hfno − 6000 Hz] + [0.01 × HNR − D] + [0.017 × H1 − H2] + [1.473 × Shim − dB] − [0.088 × Shim] − [68.295 × PSD]) × 2.9257400394.

In the present work, the ABI was computed using the Maryn and Barsties praat script [35].

### 3.7. Respiration

Data collected from the two respiratory effort transducers (RETt and RETa) were exported as .wav files. As data recording was commenced simultaneously across data recording software, the above .wav files had identical timestamps with recorded data for the Microphone and EGG signals. All four sensor signals were loaded in four channels of a new Audacity project. Actively generated common synchronization events revealed adequate initial sensor data alignment, as well as negligible (for present study purposes) clock drift at post-trials event time. For respiratory analysis the EGG signal track was not used and was therefore temporarily disregarded. Failed trials (stopped or repeated by participant’s own volition) were also disregarded and only one trial per condition was included in analysis. Valid trials were marked across sensors and labeled according to trial experimental condition. Following Salomoni et al. [22], respiratory cycles were identified visually by local maximum and minimum values of RETt and RETa data graphs to allow for optimum examination of breathing patterns.

### 3.8. Phonation Duration

Approximate phonation duration of individual trials entailing sustained both vowel and frequency was measured manually in Audacity using the selection tool and rounded to one decimal point values (sec). Glissando and arpeggio trials were excluded in order to control for in-trial pitch changes effects on phonatory duration.

## 4. Results

The above experiment data analysis revealed the results detailed below.

### 4.1. Quantitative Analysis Results

As we can see in Table 1 the average values of the pitch difference between estimations from EGG and CM signals decrease as the breathiness rate decreases, in both frequency and mel scale results. We also observe a decrease in the average CPPS values as the breathiness rates are increasing. For the non-breathy voice samples (breathiness =0 ), the average CPPS value is 19.0021, while for the excessively breathy voice samples (breathiness =4), the average CPPS value is 9.7419. We can also note that the OQ values increase as the breathiness rate increase (0.5285 for breathiness =0 and 0.6473 for breathiness =4, using the Howard algorithm) and that the use of Howard algorithm yielded higher values compared to the DECOM method. The ABI, as expected, increases as the perceptual breathiness ratings increase.

Table 2 shows the Pearson Correlation Coefficients (r) with their *p*-values (*p*) and Table 3 shows the Spearman Correlation Coefficients (ρ) with their *p*-values (*p*), for the PD, MPD, CPPS, and the OQ values with DECOM and Howard methods, as well as for the proposed multivariate index CDH and its combination with the ABI (CDH + ABI). The results demonstrate higher correlation coefficients for the CDH + ABI, CDH and ABI indexes, while the individual parameters displaying the highest significance appear to be the HOQ and the CPPS. We observe that, with the exception of PD and MPD, all correlations (CPPS, DOQ, HOQ, ABI, CDH, CDH + ABI) were statistically significant.

According to the results of the Table 1 and Table 2, the statistically significant individual parameters (CPPS, DOQ, and HOQ) were selected and a linear regression model was created. The resulting equation of the proposed index (CDH), according to the coefficients of the regression, was:

CDH = (−0.20688984 × CPPS) + (−3.21878076 × DOQ) + (9.56627174 × HOQ). Adding the ABI values, yielded the equation:

CDH + ABI = (−0.06959419 × CPPS) + (−3.26429585 × DOQ) + (8.27435347 × HOQ) + (0.2633933 × ABI).

**Figure 6:**
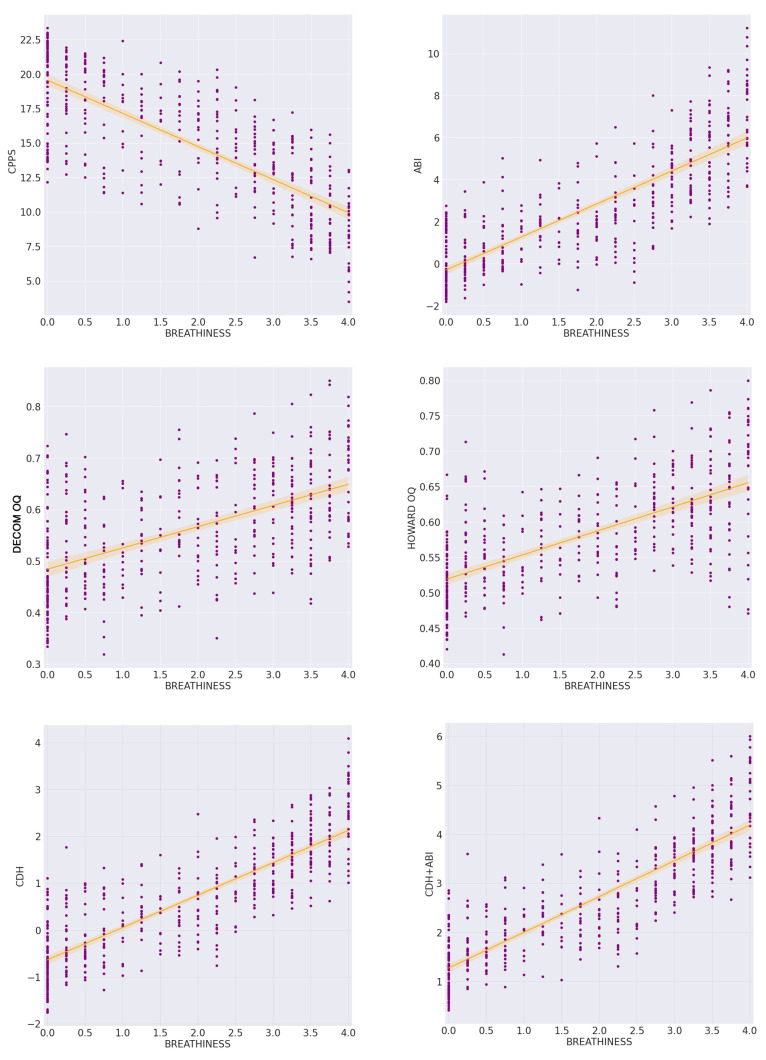
The scatter plots between average breathiness perceptual ratings and the selected acoustic measures: CPP (r = −0.7655, *p*$<10^{-5}$, ρ = −0.7647, *p*$<10^{-5}$), ABI (r = 0.8107, *p*$<10^{-5}$, ρ = 0.8279, *p*$<10^{-5}$), DOQ (r = 0.5516, *p*$<10^{-5}$, ρ = 0.5494, *p*$<10^{-5}$), HOQ (r = 0.6423, *p*$<10^{-5}$, ρ = 0.6447, *p*$<10^{-5}$), CDH (r = 0.8308, *p*$<10^{-5}$, ρ = 0.8410, *p*$<10^{-5}$), CHD + ABI (r = 0.8534, *p*$<10^{-5}$, ρ = 0.8700, *p*$<10^{-5}$).

The regression lines between breathiness perceptual ratings and the selected parameters (CPP, ABI, DOQ, and HOQ), and the indexes CDH and CHD+ABI, are shown in Figure 6.

**Figure 7:**
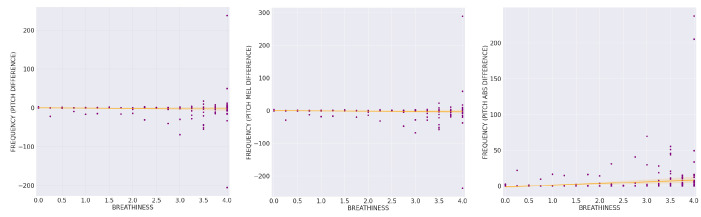
The scatter plots between average breathiness perceptual ratings and the difference of the pitch detection algorithm between the CM and the EGG signals (PD), with r = −0.0606, *p* = 0.1966, and ρ = −0.1436, *p* = 0.0021, their difference converted in mel scale (MPD) with r = −0.0494, *p* = 0.2927, and ρ = −0.1427, *p* = 0.0023, and the absolute value of their difference.

Figure 7 shows the regression lines between breathiness perceptual ratings and the difference of the pitch detection algorithm between the CM and the EGG signals (PD) with r = −0.0606, *p* = 0.1966, and ρ = −0.1436, *p* = 0.0021, their difference converted in mel scale (MPD) with r = −0.0494, *p* = 0.2927, and ρ = −0.1427, *p* = 0.0023, and the absolute value (ABS) of their difference (Table 2 and Table 3).

As we observe in Figure 7, absolute pitch difference between estimations from the CM and the EGG signals increased especially in the highest perceptual breathiness degrees. However, this pitch difference was not steadily either positive or negative. In other words, we regard that neither one of the two signals (CM and EGG) gave consistently higher or lower pitch estimates than the other.

Additionally, for the purpose of further illuminating the above relation between pitch estimation from the two signals in increasing breathiness degrees, the descriptive outcomes regarding mean absolute values of pitch difference per breathiness class in Hertz are adduced: 0.2521 (class 0), 0.7418 (class 1), 1.2193 (class 2), 2.3563 (class 3), 10.4701 (class 4). Similarly, mean values of the absolute PD as expressed in the mel scale are presented here as an indicator of the perceptual significance between the two estimation methods: 0.4020 (class 0), 1.1838 (class 1), 1.9354 (class 2), 3.6945 (class 3), 15.6859 (class 4).

### 4.2. Respiration

Audacity wave tracks containing the trials sensor data, as described in Section 3.7, were studied and analyzed visually, in order to attain information on each participant’s individual respiratory management strategies and to regard any discernible correlations to distinct trial conditions.

**Figure 8:**
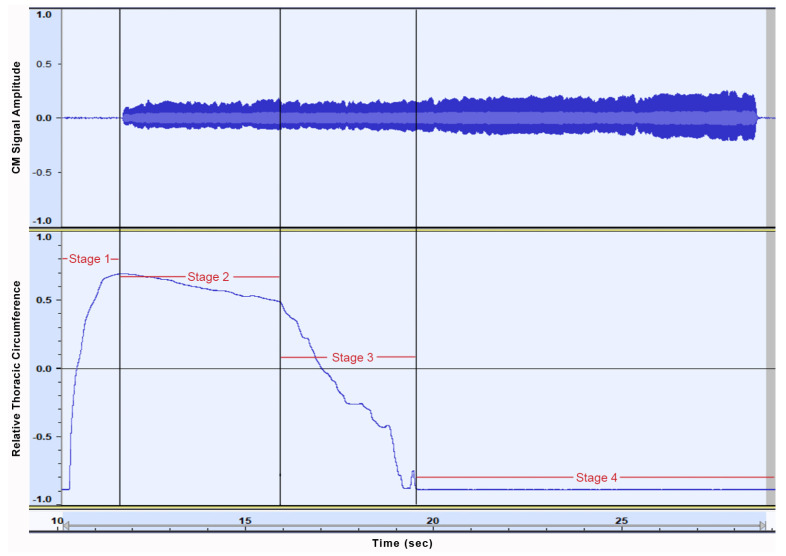
Graphic representation for the four distinct stages for thoracic movement during a typical phonation trial for Participant 1 (P1). Representations shown are for Condencer Microphone (CM) signal (**top**), and for thoracic region Respiratory Effort Transducer (RETt) (**bottom**).

After studying sensor data graphs for CM, RETt, and RETa, and conducting an observatory comparison between trials, as well as between participants P1 and P2, the following patterns seem to emerge and should be taken into consideration for further investigation. For P1 an overall uniform breathing strategy was observed across trial conditions. RETt data review revealed a thoracic movement tactic that can be visually segmented into four stages (as depicted in Figure 8): (a) a thoracic region expansion, reaching a local maximum before phonation onset (stage one), (b) a slow, linear thoracic volume decrease, leading up to approximately the midpoint of trial phonation duration (stage two), (c) a consecutive significantly more rapid volume collapse (stage three), and (d) a stabilization at a local minimum briefly after stage three (stage four).

During BSV trials, stage two of the above observations was consistently close to visual assimilation with stage three, pointing to a faster air depletion rate. This is also corroborated by mean trial durations, which were shorter in breathy phonation trials. Additionally, trials involving phonation on a high frequency seem to demonstrate a tendency for increased duration of stages two and three and thus a relatively later commencement of stage four.

**Figure 9:**
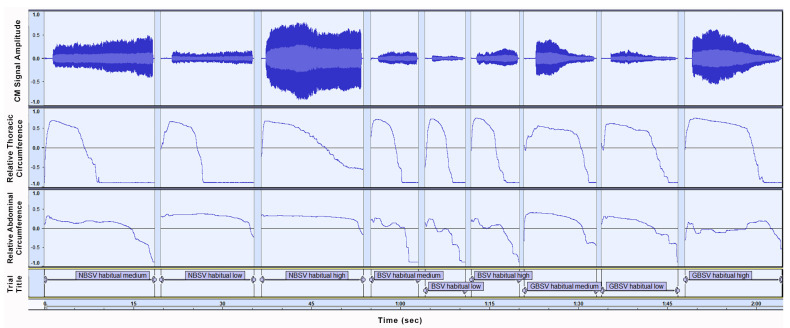
Graph of the nine initial trials for Participant 1 (P1). Sensors depicted are Microphone (top graph), Thoracic circumference Respiratory Effort Transducer (RETt) (middle graph), Abdominal circumference Respiratory Effort Transducer (RETta) (bottom graph). Individual trial labels correspond to trial conditions, namely (**left** to **right**) (1) breathiness degree: Non-Breathy singing Voice (NBSV), Breathy singing Voice (BSV), and ‘Gradual’ Breathiness singing Voice (GBSV), (2) frequency: ‘habitual’ refers to participant habitual voice frequency, (3) vocal intensity: medium, low, high.

RETa data for P1 showed phonation onset also on a local maximum for abdominal circumference expansion, revealing a full inspiratory capacity utilization. However, in contrast to evidence on thoracic region behavior, RETa data suggest a distinct abdominal kinetic strategy for phonation trials that varied with different breathiness degrees. More specifically, P1 showed a tendency to retain abdominal region at near maximum expansion levels for the greater part of phonation duration in trials on NBSV, and a minimal-to-no abdominal region volume decrease when approximating phonation ending. This strategy was altered in BSV trials to display an abdominal circumference attenuation shortly after phonation commencement, which continued during phonation and reached a much lower value than in NBSV trials. In GBSV trials, P1 employed a strategy ranging between the previous two, as can be seen in Figure 9.

**Figure 10:**
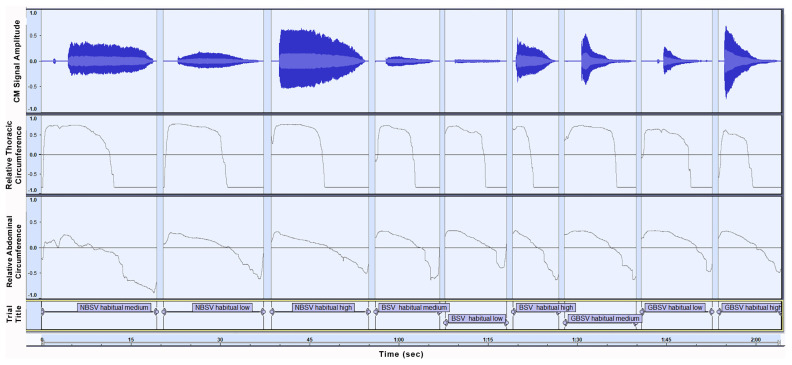
Graph of the nine initial trials for Participant 2 (P2). Sensors and trials illustrated are as described in corresponding Figure 9 for P1.

P2 also demonstrated a personal, consistent breathing kinetic strategy, as shown in Figure 10. RETt revealed a thoracic muscle behavior generally similar to that of P1, differentiated mainly by a significantly slower thoracic volume decrease during phonation stage two. Additionally, P1 did not seem to employ a distinct approach for BSV, regarding thoracic muscle activation. RETa for P2 suggest a variation of abdominal kinetic behavior when singing trial included high frequency tones, which involved a faster inward abdominal movement with a perceptually wider displacement range. Trial breathiness degree did not seem to have an obvious effect on P2 RETa waveforms.

### 4.3. Breathiness Effect on Phonation Duration

Mean phonation duration values (in seconds) for distinct breathiness level trials were NBSV = 12.43, GBSV = 9.43, BSV = 7.11.

## 5. Discussion

The assessment study of the system-in-development described in the present work yielded the results presented within the previous sections.

### 5.1. System Evaluation

An overall evaluation of the current system revealed it to be the basis for a versatile modular tool, suitable for studio and field experimentation on the singing voice. Such a tool could also be applicable to personalized vocal education and rehabilitation. All included sensors are non-invasive, and experiment participants reported to have experienced no hindrance in singing and felt free to sing normally. Equipment and settings were selected such as to produce minimum noise and data synchronization was inherently sufficient. The system was able to provide data from all three major parts of the vocal mechanism that can be analyzed and correlated to further elucidate facts regarding the singing voice.

### 5.2. Breathiness

The breathiness characteristic was studied in relation with glottal and acoustic measures. Participants’ glottal behavior, as monitored by the EGG sensor, confirmed the hypothesis for an increased vocal folds open quotient (or “quasi open quotient” (QOQ), as Herbst [56] suggests) in breathy voice prevalence samples. This is corroborated by high values of the ABI, low CPPS values, shorter phonation duration, and BSV trials breathing muscles gestures that seem to indicate a faster inhaled air depletion. All these factors indicate agreement with previously reported highly increased transglottal airflow in breathy vowel phonation [54]. Correlation between perceptual breathiness degree and each of the selected (acoustic, glottal, and combinatory model) parameters is illustrated in Figure 6, through depiction of the respective correlation analysis scatter plots. The combination of the ABI index together with the CDH index (which added EGG input data and readjusted the CPPS regression coefficient) gave the highest correlation coefficient for our dataset (r = 0.8534, *p* $<10^{-5}$, ρ = 0.8700, *p*$<10^{-5}$). Note that merely one of the variables (CPPS) used by the ABI index, when combined with two OQ extraction methods, appeared to have a strong correlation for predicting breathiness as a singing voice quality characteristic (r = 0.8308, *p*$<10^{-5}$ρ = 0.8410, *p*$<10^{-5}$). Furthermore, as expected, the ABI index showed a stronger correlation (r = 0.8107, ρ = 0.8279) than each of the selected parameters (PD, MDP, CPPS, DOQ, HOQ) independently.

### 5.3. Fundamental Frequency Estimation

Fundamental frequency estimation deviation analysis between CM and EGG signals seems to corroborate previous studies results, comparing fundamental frequency estimation between microphone and contact sensors (accelerometres and piezoelectric contact microphone) in singing [23,81] and speech [82]. Our study confirmed “comparable results” [23] to be valid also in the comparison case between CM and EGG in normophonic (NBSV) singing voice and examined the special case of BSV, specifically their difference in frequency (PD) and mel scale (MPD). In the case of BSV, our initial hypothesis was disproved, as practically no significant deviation was detected between pitch estimation with CM and EGG signal in most degrees of vocal breathiness (the mean of PD absolute values were <=1.2193 Hz for breathiness class ratings 0–2).

We observe that, based on the Pearson correlation coefficient, the correlation between breathiness and PD or MPD is statistically insignificant. The significant, albeit weak, correlation emerging from the Spearman correlation coefficient could be attributed to systematical outliers, while otherwise observation of Figure 7 indicates that these variables seem to be independent for our dataset. This result could support a claim of microphone sufficiency for pitch estimation purposes, even when measuring populations of subjects with varying degrees of vocal breathiness, thus allowing its use in the ASMA vocal tuner tool. This vocal tuner is being designed for singing voice use in Elementary School Classes. Children of about this age range (6–10) have been reported to display mean fundamental frequencies of 262 Hz for boys and 281 Hz for girls, across speaking and singing tasks, with a tendency for higher frequencies during singing tasks [83]. For a voice rated with a class 2 breathiness (of the described 0–4 classification), the absolute mean difference between CM and EGG pitch estimation was found to be 1.2193 Hz. Even in such a case this pitch deviation in a mean frequency of singing around 270 Hz would be the perceivable analogue of 0.077 semitones (7.7 Cents), while in a comfortable (for children) tone of 350 Hz, estimation difference in a mildly breathy voice (class 1) would have a mean of 0.7418 Hz, meaning 0.035 semitones (3.5 Cents). In his chapter “The Perception of Singing” [84], Sundberg describes a 2.9 cent interval at 300 Hz as “impossible for almost any listener to detect under any experimental conditions”, and states that “the difference limen for frequency is at least 6 cents but may be considerably higher” [84]. This further supports the validity of our sensor (CM) and algorithm choice for the task at hand.

### 5.4. Respiration

Professional singers have been known to employ distinct respiratory management strategies. P1 and P2 have displayed differences in abdominal kinetic behavior during NBSV trial. However, when faced with a need for air added to the voice (BSV trials), they both opted to use an inward movement of the abdominal muscles, to apparently force an upward diaphragmatic movement and elevate subglottal air pressure. P1 and P2 RET analysis agreed with general expectation for experienced singers with years of respiratory training and indicated consistent (albeit distinct between participants) individual respiratory management strategies. However, qualitative analysis pointed to evidence of variation in extreme vocal breathiness conditions and high frequency phonation. These evidence are not generalizable, as the experiment was performed as a case study and involved intentionally breathy phonation (air added to the voice [32]) by trained professionals, and not untrained participants with inadvertently breathy or pathological voices. Nevertheless, this analysis can be used as a showcase of the proposed multi-sensor voice analysis system capability, while more cases could be the subject of further experimentation.

### 5.5. Study Limitations and Future Work

The system presented here is a work-in-progress towards a modular multi-sensor tool for singing voice analysis. A dedicated software that will record and automatically synchronize the data from all sensors (without the required use of third party or sensor manufacturer’s programs) is currently in discussion. Additionally, the Kinect Azure SDK [85] is already being implemented to the system, using skeletal tracking [86] as a means of monitoring and recording (a) singers’ postural alignment, and (b) kinetic habits connected to, or affecting, the production of the singing voice. This camera model has increased portability, and has been reported to have higher accuracy and lower noise than previous models, and operate in various distinct modes (e.g., different fields of view) [87]. Another possible sensor addition to this modular system is that of an accelerometer. This has been tested for the estimation of vocal measures such as fundamental frequency, glottal airflow, open quotient [27], and subglottal pressure [28].

In the present study, we have provided a brief qualitative analysis of the respiratory measurements obtained from the evaluation setup described in Section 3.7 and Section 4.2. In a future work, we will provide quantitative data analyses of the outcomes from more experiments than the proof of concept results presented herein. The volume of both experimental and analytical work needed to obtain meaningful outcomes presumes a venture by far surpassing the scope of the work we have presented here. Related data have been assayed in a singer’s breathing dedicated study using Principal Component Analysis (PCA) [22] and it is our intention to use similar protocols and fitting techniques for analysis, as well as appropriate low-pass filtering to eliminate high frequency instrument noise.

COVID-19-related restrictions and precaution measures led to the limitation of the current experiment’s accessible and willing participant number, as the study design already required professional singers demonstrating a high expertise level in vocal control. Therefore, the experimental part of this work served mostly as a case study to assess the current system stage, and to extract predictive results regarding vocal breathiness, with professionals intentionally adding air to their voice.

Apart from the testing a stage of this in-development system, potential innovative aspects of the presented work are (i) the proposed CDH multivariate model and its combination with the ABI model, which best predicted the perceived breathiness rating for our dataset, and (ii) the comparison of pitch detection algorithm results between microphone and EGG signals in vocal breathiness conditions. Testing the above models and results in larger data pools has been planned.

Further extensions of the current work would also pertain to the existing dataset expansion with (i) greater participant and judges populations; (ii) participant groups of distinct vocal proficiency levels and ages; and (iii) inclusion of a number of categorized perceptual vocal features, related to voice quality, and especially vocal technique and idioms. Our goal for the integrated form of the system is for it to support multivariate correlations, using machine learning techniques and models, as well as real-time analysis and voice monitoring.

## Figures and Tables

**Table 1:** Descriptive outcomes: Average values for different perceptual ratings of breathiness. BR: breathiness rating, PD: Pitch Difference, MPD: Pitch Difference in Mel scale, CPPS: Smoothed Cepstral Peak Prominence, DOQ: Open Quotient with DECOM method, HOQ: Open Quotient with Howard method.

BR	PD	MPD	CPPS	DOQ	HOQ	ABI
0	−0.1197	−0.1624	19.0021	0.4969	0.5285	0.1542
1	−0.5997	−0.6998	16.6308	0.5119	0.5483	1.4276
2	−1.0749	−1.1693	15.7304	0.5652	0.5778	2.0935
3	−2.0878	−2.1259	13.2119	0.6067	0.6225	3.8265
4	−2.9783	−2.9235	9.7419	0.6391	0.6473	6.1339

**Table 2:** Pearson Correlation Coefficients, r (*p*-values), between the breathiness rating and the selected parameters (PD: Pitch Difference, MPD: Pitch Difference in Mel scale, CPPS: Smoothed Cepstral Peak Prominence, DOQ: Open Quotient with DECOM method, HOQ: Open Quotient with Howard method, CDH: CPPS + DOQ + HOQ, CDH + ABI: CPPS + DOQ + HOQ + ABI).

	PD	MPD	CPPS	DOQ	HOQ	ABI	CDH	CDH + ABI
r	−0.0606	−0.0494	−0.7655	0.5516	0.6423	0.8107	0.8308	0.8534
*p*	0.1966	0.2927	<$10^{-5}$	<$10^{-5}$	<$10^{-5}$	<$10^{-5}$	<$10^{-5}$	<$10^{-5}$

**Table 3:** Spearman Correlation Coefficients, ρ (*p*-Values). BR: breathiness rating, PD: Pitch Difference, MPD: Pitch Difference in Mel scale, CPPS: Cepstral Peak Prominence, DOQ: Open Quotient with DECOM method, HOQ: Open Quotient with Howard method, CDH: CPPS + DOQ + HOQ, CDH + ABI: CPPS + DOQ + HOQ + ABI).

	PD	MPD	CPPS	DOQ	HOQ	ABI	CDH	CDH + ABI
ρ	−0.1436	−0.1427	−0.7647	0.5494	0.6447	0.8279	0.8410	0.8700
*p*	0.0021	0.0023	<$10^{-5}$	<$10^{-5}$	<$10^{-5}$	<$10^{-5}$	<$10^{-5}$	<$10^{-5}$

## Data Availability

Data available in https://github.com/nataliakotsani/Singing-Voice-Multi-Sensor-Analysis-Tool/, https://doi.org/10.5281/zenodo.5732933.
